# Role of Neuronal NADPH Oxidase 1 in the Peri-Infarct Regions after Stroke

**DOI:** 10.1371/journal.pone.0116814

**Published:** 2015-01-24

**Authors:** Dong-Hee Choi, Ji-Hye Kim, Kyoung-Hee Lee, Hahn-Young Kim, Yoon-Seong Kim, Wahn Soo Choi, Jongmin Lee

**Affiliations:** 1 Department of Medical Science, Konkuk University School of Medicine, Seoul, Republic of Korea; 2 Department of Rehabilitation Medicine, Konkuk University School of Medicine, Seoul, Korea; 3 Center for Neuroscience Research, Institute of Biomedical Science and Technology, Konkuk University, Seoul, Korea; 4 Burnett School of Biomedical Sciences, College of Medicine, University of Central Florida, Orlando, FL 32827, United States of America; 5 Department of Immunology and Physiology, Functional Genomics Institute, College of Medicine, Konkuk University, Chungju, Korea; School of Pharmacy, Texas Tech University HSC, UNITED STATES

## Abstract

The molecular mechanism underlying the selective vulnerability of neurons to oxidative damage caused by ischemia—reperfusion (I/R) injury remains unknown. We sought to determine the role of NADPH oxidase 1 (Nox1) in cerebral I/R-induced brain injury and survival of newborn cells in the ischemic injured region. Male Wistar rats were subjected to 90 min middle cerebral artery occlusion (MCAO) followed by reperfusion. After reperfusion, infarction size, level of superoxide and 8-hydroxy-2′-deoxyguanosine (8-oxo-2dG), and Nox1 immunoreactivity were determined. RNAi-mediated knockdown of Nox1 was used to investigate the role of Nox1 in I/R-induced oxidative damage, neuronal death, motor function recovery, and ischemic neurogenesis. After I/R, Nox1 expression and 8-oxo-2dG immunoreactivity was increased in cortical neurons of the peri-infarct regions. Both infarction size and neuronal death in I/R injury were significantly reduced by adeno-associated virus (AAV)-mediated transduction of Nox1 short hairpin RNA (shRNA). AAV-mediated Nox1 knockdown enhanced functional recovery after MCAO. The level of survival and differentiation of newborn cells in the peri-infarct regions were increased by Nox1 inhibition. Our data suggest that Nox-1 may be responsible for oxidative damage to DNA, subsequent cortical neuronal degeneration, functional recovery, and regulation of ischemic neurogenesis in the peri-infarct regions after stroke.

## Introduction

Cerebral ischemia occurs because of a local reduction or an arrest of blood supply, and can lead to neuronal cell death in the ischemic region. Focal cerebral ischemia is the most common subtype of clinical stroke. Since the establishment of middle cerebral artery occlusion (MCAO)-reperfusion, in rats as an animal model of focal ischemia [1], this model has been widely used to study injury and recovery after stroke [2]. The pathophysiological mechanisms of the ischemia—reperfusion (I/R) injury are complex. Cell death progresses within minutes in the ischemic core, whereas in the peripheral zones of the infarct, which are perfused by collateral blood vessels, active cell death mechanisms are recruited and proceed more slowly, rendering the peri-infarct tissue more likely to be rescued by pharmacological treatment or modulation of circumstances [3]. Molecular mechanisms leading to cell death during ischemic brain injury include excitotoxicity and ionic imbalance, free radical stress, and apoptotic-like cell demise [3, 4].

NADPH oxidases (Nox) play an important role in I/R tissue injury of various organs, including the brain. Indeed, under certain conditions, Nox proteins can generate large amounts of reactive oxygen species (ROS), and several enzymes of this protein family and their cytosolic activator proteins are expressed in the central nervous system [5]. Nox has been shown to play a significant role in I/R-induced brain injury through superoxide production. The family of Nox proteins is currently known to consist of seven members. Nox1, Nox2 (previously termed gp91phox), and Nox4 are expressed under physiological conditions in the central nervous system, including in intracranial vessels and neuronal tissues [5–7]. The role of Nox2 in I/R-induced brain injury has been extensively studied [6–8]. A role for Nox4 in infarcted brain tissue after I/R injury has been demonstrated [9, 10]. The higher expression of Nox1 and Nox4 compared with Nox2 in endothelial cells of cerebral arteries [5] suggests a prominent function for these enzymes in the cerebral vasculature. Few studies have addressed the role of Nox1 in experimental stroke. A moderate reduction in lesion volume and cerebral edema, and an ameliorated blood—brain barrier (BBB) leakage with relatively mild ischemic damage (60 min ischemia followed by 23 h of reperfusion) was observed after knockout of Nox1 [9]. However, a lack of Nox1 was found not to confer protection in transient [9, 11, 12] or permanent [9] cerebral ischemia. Therefore, the precise role of Nox1 in I/R injury is controversial and remains unknown.

Stroke enhances subventricular zone (SVZ) cell proliferation, and that these cells can differentiate into mature striatal neurons and replace damaged neurons [7, 12]. However, the vast majority of neuroblasts die primarily by apoptotic mechanisms, and only a small population of newly generated neurons survive beyond 6 weeks (w) after stroke [13–16]. The reasons why these death-promoting events are activated are not known. However, because the environment of newly arrived cells must be receptive if they are to survive, providing trophic support and eventually leading to their integration within the tissue [16], many experimental trials have been undertaken to enhance endogenous neurogenesis after stroke [5, 6, 9, 12]. Therefore, in order to find a microenvironment promoting the survival of newborn cells in ischemic neurogenesis, we determined the role of Nox1 in I/R-induced oxidative damage and the effect of Nox1 on the survival of newborn cells in ischemic neurogenesis after stroke.

## Materials and Methods

### Animals

A total of 128 Male Wistar rats (8 weeks, weighing 291 + 2.57 g; Samtako BioKorea. Co. Ltd., Korea; 44 sham control rats and 84 MCAO operated rats) were enrolled in this study. Animals were singly housed in smooth bottomed plastic cages (28 W X 42 D X 20 H (cm)) with beta chip bedding (Agrolab group, Bavaria, Germany) in a specific pathogen free room maintained on a 12-h light/dark cycle. Food and water were available ad libitum. The room temperature was maintained at 22°C. The room was illuminated by incandescent lamps (luminous flux, 11.77lm). In order to accustom the animals to the laboratory environment, an acclimation period of 2 weeks was allowed before the initiation of the experiment.

**Fig 1:**
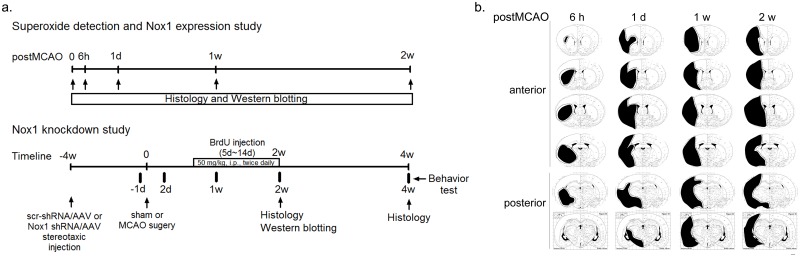
The timeline of the experiment and schematic diagrams of the infarct core and the peri-infarct region after MCAO (a) Rats submitted to sham operation or MCAO. Sham operated rat (SC) and MCAO rats were used for superoxide detection, protein expression studies, and immunohistochemical analysis at 6 hours (6 h), 1 day (1 d), 1 week (1 w), and 2 weeks (2 w) after MCAO. In Nox1 knockdown study, AAV particles containing either Nox1 shRNA/AAV or scramble shRNA/AAV (Scr-shRNA/AAV) were stereotaxically injected into the cortex and striatum at 4 weeks before the MCAO or sham-operation. SC and MCAO rats received an injection of the cell proliferation marker BrdU (50 mg/kg, i.p., twice daily) from days 5 to 14 after sham or MCAO operation. All animals were used for behavioral analysis, histological study and protein expression study. (b) Schematic diagrams of the infarct core and the peri-infarct region delineated by Nissl staining at 6 h, 1 d, 1 w, and 2 w after MCAO—reperfusion (n = 4/group). Black: infarct core; black dotted line: peri-infarct region. Scale bar = 1 mm.

Animal treatments including anesthesia and euthanasia were carried out in accordance with the Principle of Laboratory Animal Care (NIH publication No. 85–23, revised 1985). All experimental procedures were approved by the Animal Experiment Review Board of Laboratory Animal Research Center of Konkuk University (KU10065). They are in accordance with the Stroke Therapy Academic Industry Roundtable (STAIR) criteria [17] for preclinical stroke investigations. Detailed timeline for experiment were described (Fig. 1a).

### Rat model of focal cerebral ischemia and reperfusion

Rats were anesthetized with isoflurane (4% for surgical induction, 1.75% for maintenance) in NO_2_: O_2_ (70%:30%) during surgical procedures. Body temperature was monitored continuously with a rectal probe and maintained at 37.5°C ± 0.5°C using a heating pad. MCAO model was established by proximal occlusion of the right middle cerebral artery with the use of a 4–0 silicone-coated nylon monofilament (5 mm length of the coated end with a diameter of 0.35 mm) [18]. After 90-min occlusion, reperfusion was accomplished by careful withdrawal of the monofilament and the rats were returned to their cages.

In Nox1 expression study, among the 48 rats, 40 of them underwent middle cerebral artery occlusion-reperfusion (MCAO-r) surgery and the 32 survivors were equally distributed into the MCAO-6 h (hours), the MCAO-1 d (day), the MCAO-1 w (week), and the MCAO-2 w (weeks) groups, as indicated below. Thus, the survival rate after MCAO-r, comprising the rats of MCAO-6 h, the MCAO-1 d, the MCAO-1 w, and the MCAO-2 w groups, was 80.0%. Consequently, the remaining 32 animals were randomly assigned to the following 5 groups: 1) MCAO-6 h (n = 8), 2) MCAO-1 d group (n = 8) 3) MCAO-1 w group (n = 8), 4) MCAO-2 w group, 5) Sham control group (SC, n = 8). SCs were treated similarly to the operated rats except the MCAO. After the operation, rats were kept in an animal resource facility with food and water ad libitum. Four rats for histological analysis and 4 rats for Western blot analysis were used.

In Nox1 knockdown study, 80 rats were subjected to stereotaxic injection of AAV particles containing either Nox1 shRNA/AAV (Nox1 shRNA, n = 40) or scrable shRNA /AAV (scr-shRNA n = 40) into the cortex and striatum. After 4 weeks, 22 rats of the 40 Nox1 shRNA-injected rats and 22 rats of the 40 scr-shRNA-injected rats underwent MCAO-reperfusion (MCAO-r) surgery and the 36 survivors (survival rate = 82.3%) and 36 sham control rats were equally distributed into the animals were randomized in four different groups using 2-by-2 table of MCAO or shRNA/AAV, including 1) sham operated scr-shRNA (n = 18), 2) sham operated Nox1 shRNA (n = 18), 3) MCAO operated scr-shRNA (n = 18), and 4) MCAO operated Nox1 shRNA (n = 18). Animals were sacrificed at 2 weeks (n = 10/group) and 4 weeks after MCAO-reperfusion (n = 8/group). Animals were number-coded and investigators were always blinded to the treatment groups until the end of the data analysis. Occlusion and reperfusion were verified in each animal by laser Doppler flowmetry (Moor Instruments, UK).

### *In situ* visualization of superoxide

The spatial production of O_2_^−^ during cerebral ischemia was investigated by the *in situ* detection of oxidized hydroethidine (HEt) method as previously described [19] with minor modifications. HEt (Molecular Probes, Eugene, OR) is taken up by living cells and oxidized to a red fluorescent dye, ethidium, specifically by O_2_^−^, but not by other reactive oxygen species in the cells [20]. Briefly, on 6 h, 1 d, 1 w, or 2 w post-MCAO operation, rats were injected i.p. with 2 ml of PBS containing 1 ug/ul HEt and 1% dimethysulfoxide. Animals (n = 4/group) were sacrificed 60 min later, and the brain was removed and frozen in dry ice. The cortex and striatum sections (30 μm thick) mounted onto gelatin-coated glass slides were examined for HEt oxidation product, ethidium accumulation, by fluorescence microscopy.

### Nissl staining

Rats (n = 8/group) were deeply anesthetized ketamine (50 mg/kg) and xylazine (5 mg/kg) mixture (i.p.) and transcardially perfused with saline containing 0.5% sodium nitrite and 10 U/ml heparin sulfate, followed by cold 4% formaldehyde generated from paraformaldehyde in 0.1 M PBS (pH 7.2). Brains were post-fixed in the same solution for overnight and infiltrated with 30% sucrose overnight. Free-floating sections (40 μm) were obtained from from bregma—5.2 to 2.2 mm using a Cryostat. Sections were mounted in glass slide and then stained Nissl. Nissl staining was incubated in 0.1% Cresyl violet solution for 10 minutes at room temperature and rinsed quickly in distilled water, and dehydrated in serial diluted ethanol and cleaned in xylene. Images of the mounted sections were captured with a CCD camera (Cohu Inc., San Diego, CA, USA) mounted above a light box (Biotec-Fischer Colour Control 5000, Reiskirchin, Germany). Using this method, only viable non-infarcted tissue is stained, making the infarct area clearly visible. The infarct areas and the peri-infarct areas were indicated as a schematic diagram (Fig. 1b).

### Measurement of infarct volume

Using Nissl stained section, infarct volume (total, cortical and subcortical) was quantified using image analysis software (ImageJ v1.3, NIH), correcting for brain edema, according to the following formula: CIV = [LHA−(RHA−RIA)]×THICKNESS OF SLICE, where CIV is corrected infarct volume, LHA is left hemisphere area, RHA is right hemisphere area and RIA is right hemisphere infarct area [12]. Corrected infarct volumes and the distance between individual brain sections were then used to estimate the total infarct volume.

### Sampling procedure

Rats (n = 4/group) were deeply anesthetized ketamine (50 mg/kg) and xylazine (5 mg/kg) mixture (i.p.), and the brains were rapidly removed and placed in ice-cold saline. They were then placed on a chilled glass plate with the ventral surface uppermost, and the MCAs were identified. Three blocks of tissue, ∼1 mm thick per block, were removed by transecting the brain coronally with a razor blade bracketing the origin of the MCA at ~ 10 mm from the interaural line. Areas were sampled in each animal according to the individual distribution of the infarction, which can be easily detected with the naked eye because it is significantly whiter than the rest of the tissue. The tissue blocks were placed flat on the dish, and samples were taken with sterile forceps from the infarct area, the peri-infarct area (outer borders of the infarction), and the contralateral hemisphere homologous to the infarct area and the peri-infarct area.

### Western blot analysis

Tissues (n = 4/group) were washed with ice-cold PBS and lysed on ice in RIPA buffer (50mM Tris-HCl pH 7.4, 150mM NaCl, 1% NP40, 0.25% Na-deoxycholate, and 0.1% SDS) containing a protease inhibitor mixture and phosphatase inhibitors (Sigma-Aldrich, St. Louis, MO). Thirty μg of soluble protein was subjected to SDS-PAGE and electrotransferred onto a PVDF membrane. Specific protein bands were detected by using specific anti-Nox1 (Santa Cruz Biotechnology, Santa Cruz, CA) and anti-Nox2 (BD bioscience, San Jose, CA.) antibodies and Enhanced Chemiluminescence (Pierce, Rockford, IL).

### Cell culture

Murine aortic smooth muscle cells (SMC) taken from Wild-type and *Nox1* knockout mice (C57BL/6x129SvEv) were generously donated by Dr. Yun Soo Bae (Ewha Womans University) [19]. SMC were grown in DMEM containing 10% FBS, 100 IU/l penicillin, and 10 g/ml streptomycin at 37°C with 5% CO2 supply in humidified atmosphere. For experiments, the cells were plated on polystyrene tissue culture dishes at a density of 1×10^6^ cells/well in 6-well culture plates, or 2×10^6^ cells/100 mm plate. Experiments were performed using SMC between passages 6 and 8.

### Rac1 activation assay

1 mg of protein extracted from peri-infarct tissue (n = 4/groups) was incubated with 10 mg of agarose beads containing p21-binding domain (PBD) of the p21-activated protein kinase 1 (PAK1), an effector of activated Rac, for 1 hr at 4°C. The beads were collected by centrifugation and washed two times in the lysis buffer. The beads were resuspended in sample buffer and boiled for 5 min. Proteins were resolved by SDS-PAGE using a 10–20% Tricine gel, transferred electrophoretically and visualized using anti-rat Rac1 antibody (Cell BioLabs, Inc. CA, USA) followed by enhanced chemiluminescence. For the positive control, the nonhydrolyzable GTP analog GTPgS was used according to the manufacturer’s protocol (Cell BioLabs, INC. CA, USA).

### BrdU labeling

Neurogenesis was assessed by analyzing incorporation of BrdU (Sigma Aldrich, St. Louis, MO, USA), a thymidine analog and a marker of proliferating cells. BrdU solutions were prepared at 5 mg/mL in sterile saline with 0.007 N NaOH to obtain a 50 mg/kg dose (10 mL/kg). Sham-operated rats and rats with MCAO received an injection of the cell proliferation marker BrdU (50 mg/kg, i.p., twice daily) from days 5 to 14.

### Immunohistochemistry

For 7,8-dihydro-8-oxo-deoxyguanine (8-oxo-dG) staining, brain sections were incubated in 70% ethanol precooled to -20°C for 10 min on ice followed by 4 N HCl to denature DNA. After rinsing with PBS, the sections were soaked in 37°C PBS supplemented with 100 μg/ml DNase-free RNase A for 1 h. Sections were washed in 0.1 M PBS, incubated in 0.1 M PBS containing 5% normal goat serum and 0.3% TritonX-100 for 1 h, and subsequently incubated overnight with mouse monoclonal 8-oxo-dG (Trevigen, Gaithersburg, MD, USA, 1:1,000) antibody 4°C. The sections were then incubated with biotinylated anti-mouse IgG (Vector Lab, Burlingame, CA, USA, 1:500) for 1 h, followed by avidin/biotin/peroxidase (Vector Lab, Burlingame, CA, USA) staining for 1 h in a humidified chamber. 0.1 M PBS containing 1.5% bovine serum albumin (Sigma-Aldrich, St. Louis, MO, USA) was used to wash sections on slides between all steps. The antigen-antibody complexes were visualized by incubation for 2 min in 0.05% 3,3’-diaminobenzidine (Sigma-Aldrich, St. Louis, MO, USA) and 0.003% H_2_O_2_ (Sigma-Aldrich, St. Louis, MO, USA) and then mounted sequentially in glass slides using permanent mounting medium (Vector Lab, Burlingame,CA, USA). Mounted slices were evaluated on light microscope.

### Double fluorescent immunostaining of tissues

Free-floating sections (40 μm) were washed in 0.1 M PBS, incubated in 0.1 M PBS containing 5% normal donkey serum and 0.3% TritonX-100 for 1 h, and subsequently incubated overnight with primary antibodies (Nox1, Santa Cruz Biotechnology, Santa Cruz, CA, USA, 1:500; GFAP, Millipore, Billerica, MA, USA, 1:500; CD11b, Millipore, Billerica, MA, USA, 1:200; Ki67, Thermo Scientific, Fremont, CA, USA, 1:200; 5-bromo-2′-deoxyuridine (BrdU, Abcam, Cambridge, UK), 1:500; doublecortin (DCX), Santa Cruz Biotechnology, Santa Cruz, CA, USA, 1:500; NeuN, Millipore, Billerica, MA, USA, 1:1000; active caspase-3, Cell Signaling Technology, Inc. Danvers, MA, USA, 1:500) in 2% normal donkey serum (Vector Lab, Burlingame,CA, USA) in PBS at 4°C and incubated in a 1:200 dilution of Alexa Fluor conjugated donkey anti-rabbit (546) or donkey anti-mouse (647) antibodies (Invitrogen, Grand Island, NY, USA) for 1h at room temperature, washed with PBS, and then mounted sequentially in glass slides using Vectashield (Vector Lab, Burlingame, CA, USA). Mounted slices were evaluated for fluorescence under settings for 546 and 647 emissions on a confocal microscope (Olympus, USA).

### Establishment of U6-NOX1 shRNA-CMV-EGFP/AAV and AAV viral package

U6 promoter-driven shRNA expression system was established in AAV2 vector. EGFP expression is separately controlled by a CMV promoter as a marker for the transduction efficiency. Nox1 shRNA was designed based on the siRNA sequence (5’-CCTTTGCTTCC TTCTTGAAATCTAT-3’) which efficiently knocked down Nox1 expression in N27 cells [21]. Rat Nox1 shRNA sequence (5’-AGCTTCCTTTGCTTCCTTCTTGAAATCTATTTCAAGAGAAT AGATTTCAAGAAGGAAGC AAAGGTTTTTTG-3’) was inserted between HindIII and EcoRI sites in the U6-CMV-EGFP/AAV vector. Either empty vector or NOX1 shRNA/AAV were co-transfected with pHelper and pAAV-RC to HEK293 cells using a standard calcium phosphate method. After 72 h, the cells were harvested and crude rAAV vector solutions were obtained by repeated freeze/thaw cycles. The cleared crude lysate was then applied on a heparin-agarose column (Sigma-Aldrich, St. Louis, MO). After all the lysate went through the column, the matrix was washed twice with 25 ml of PBS (pH 7.4, 0.1M NaCl). The virus was then eluted with 15 ml of PBS (pH 7.4, 0.4M NaCl). The elutes was concentrated to about 1ml with a Millipore Centriplus YM-30 Centrifugal Filter by centrifugation 4000 rpm, 15–40 min. To adjust the NaCl concentration to physiological levels, the filter device was refilled with PBS, pH 7.4, and the virus was concentrated to 250–300 ul again. After removal of the virus-containing solution, the membrane of the filter device was washed three times with 100 ul of PBS, pH 7.4, which was added to the main part of the recombinant AAV2. The fractions containing high-titer rAAV vectors were collected and used for injection into animals. The number of rAAV genome copies was semiquantified by PCR within the CMV promoter region using primers 5’-GACGTCAATAATGACGTATG-3’ and 5’-GGTAATAGCGATGACTAATACG-3’.

### Adeno associated virus 2-mediated Nox1 knockdown

AAV particles containing either Nox1 shRNA/AAV or empty vector/AAV [21] were stereotaxically injected into the cortex and striatum for 4 weeks prior to MCAO operation. All rats were respectively allocated into 2 groups (n = 40/group), Nox1 shRNA/AAV and scramble shRNA/AAV as control groups. Rats were deeply anesthetized ketamine (50 mg/kg) and xylazine (5 mg/kg) mixture (i.p.) and placed in a rat stereotaxic apparatus, two sites in the right cortex [coordinate: anteroposterior (AP), –0.4 mm; mediolateral (ML), + 2.0 mm; dorsoventral (DV), –1.5 mm] and striatum [coordinate: AP, –0.4 mm; ML, + 2.0 mm; DV, –5.0 mm] that are predicted to become the peri-infarct regions at 2 weeks after MCAO surgery were selected to inject Nox1 shRNA/AAV and scramble shRNA/AAV, respectively, according to the grouping. A total of 1x10^11^ genome copy/ml recombinant AAV particles encoding shNox1, or empty vector diluted in 2 μl ice-cold sterilized phosphate buffered saline (PBS) were used in every animal. The injection rate was 0.5 μl/min, and the syringe was kept in place for an additional 5 min before being retracted slowly. Rats were subjected to sham or MCAO surgery after 4 weeks. Animals were sacrificed at 2 weeks (n = 10/group) and 4 weeks after MCAO-reperfusion (n = 8/group). Tissue sections and protein extractions taken from brain tissues of SC and MCAO animals were used in histological analysis (n = 6~8/group) and Western blot analysis (n = 4/group), respectively.

### Quantitative analysis

Sections including the cortex and striatum from 4~6 rats per group were subjected to analysis. Five regions of interests (ROIs) of 0.1mm^2^ per one section in the peri-infarct areas (bregma -0.4 to 2 mm; 6 sections per rat, every fifth sections) were selected. The number of DHE-, NeuN-, 8-oxo-dG-, Nox1-, BrdU-, Ki67-, or DCX-positive cells were counted in each ROI and averaged. Data represented as percentage of total cell. All quantitative analyses were carried out in a blind manner.

### The parallel bar walking test

Parallel bar testing (n = 8/group) was particularly sensitive to the hindlimb coordination impairment [22]. The parallel bar apparatus consisted of two parallel wooden rods (1.0 cm diameter each, 100 cm long), with an inter-rod distance of 2.5 cm, connected to platforms at each end (15 x 50 cm^2^). The number of times that the subject placed two hind paws on one rod, dropped a hind paw below the rod, or fell or swung under the rods was recorded. Number of errors made per meter in 1 min were calculated and scored. The graded scoring system ranges from 0 to 5 depending on the motor function behavior.

0–5 point grading scale

0, No error;

1, Less than 1 error made within 1 min per meter length;

2, Equal or more than one error made within 1 min per meter length;

3, More than two error made within 1 min per meter length;

4, More than 3.5 error made within 1 min per meter length;

5, More than six errors or cannot traverse.

### Data analysis and statistics

Data are expressed as percentages of values obtained in control conditions, and are presented as mean ± S.E.M. Statistical analysis between two groups was assessed using a two-tailed, unpaired Student’s *t* test. Differences among three, four, or five groups were analyzed using a one-way ANOVA followed by Newman-Keuls Multiple Comparison Test. The parallel bar walking test were analyzed using a two-way repeated measures analysis of variance (ANOVA), followed by a post hoc least significant differences multiple comparisons test. Values of *P* < 0.05 were considered significant. All data analyses were performed using SPSS version 20.0 software.

## Results

### Increase in superoxide generation and DNA oxidation in the brain after ischemia—reperfusion injury

**Fig 2:**
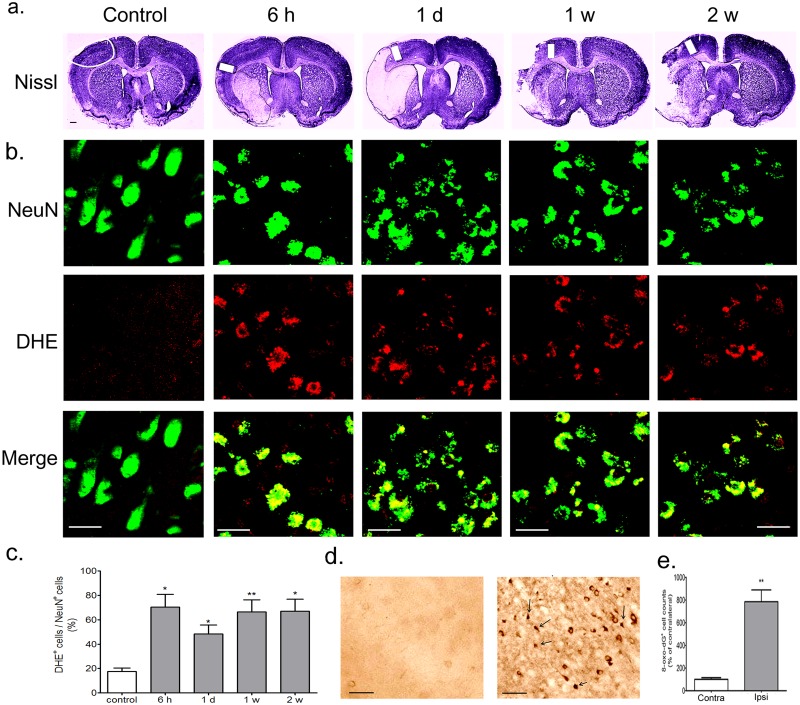
Superoxide generation and DNA oxidation increased in the ischemia—reperfusion brain. (a) Representative photomicrograph of Nissl staining showing infarct region in ischemia—reperfusion brain for at 6 h, 1 d, 1w, and 2 w. Scale bars = 1 mm. White solid line and white colored boxes indicate the peri-infarct region in the cortex showing DHE staining. (b) Representative photomicrograph of NeuN (green) and DHE (red) staining showing superoxide levels in neurons of ischemia—reperfusion brain for 6 h, 1 d, 1 w, and 2 w. Superoxide in neurons is demonstrated as yellow staining after merging green (NeuN) and red (DHE) images. Scale bars = 20 μm. (c) DHE-stained cells of the NeuN-stained cells were counted in the peri-infarct region. Results are presented as the mean ± SEM, n = 4/4. *p<0.05, **p<0.01 vs control. (d) Representative photomicrograph of 8-oxo-2dG immunostaining showing DNA oxidation levels in ischemia—reperfusion brain at 2 weeks. Left panel is contralateral region in the cortex. Right panel is cortical peri-infarct region. Black arrows indicate 8-oxo-2dG-staining in the nucleus. Scale bars = 30 μm. (e) 8-oxo-2dG-stained cells were counted in the ischemia—reperfusion brain. Results are presented as the mean ± SEM, n = 4/4. **p<0.01 vs the contralateral region. Contra: contralateral; Ipsi: ipsilateral.

Cresyl violet staining in the lateral part of the striatum disappeared at 6 hours (h) of reperfusion after 90 min of MCAO. Areas of infarction were noted in the striatum and in MCA-related areas of the parietal cortex. The extent of infarction widened further at 1 day (d), 1 week (w), and 2 w after reperfusion to include the entire MCA territory in the parietal cortex. Representations of the extent of the infarct area and peri-infarct regions are shown in Fig. 1b and Fig. 2a.

Generation of superoxide was observed in the peri-infarct regions (Fig. 2a) of rats with MCAO—reperfusion injury. Superoxide levels increased significantly in NeuN-positive neurons of the peri-infarct regions at 6 h, 1 d, 1 w, and 2 w after MCAO—reperfusion (Fig. 2b and c). Some NeuN-positive cells displayed irregular morphology in the peri-infarct region at 1 d, 1 w, and 2 w after MCAO—reperfusion.

DNA oxidation also increased in the peri-infarct regions at 2 w after MCAO—reperfusion (Fig. 2d and e).

### Increased Nox1 expression after MCAO—reperfusion in neurons of the peri-infarct regions

**Fig 3:**
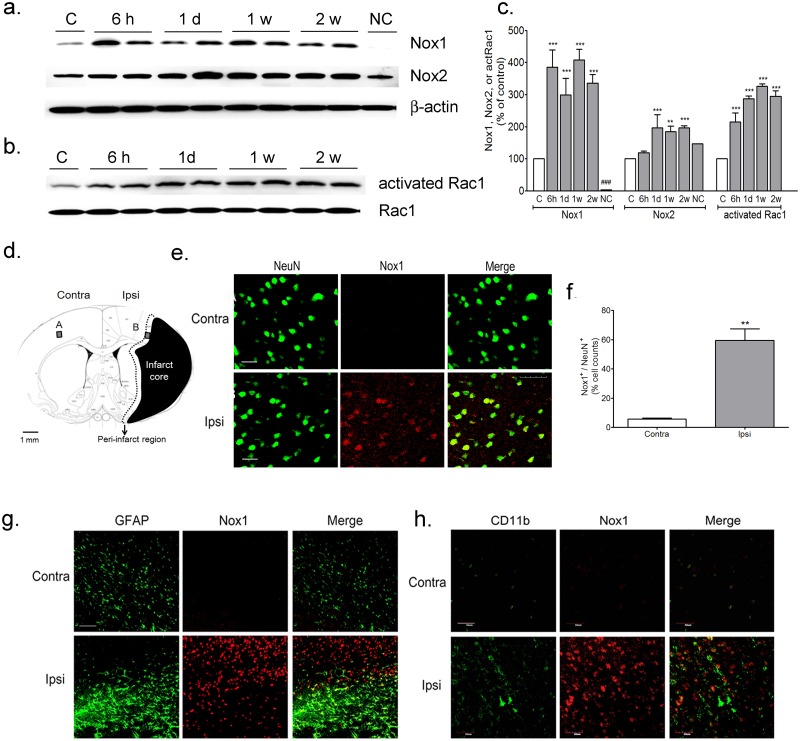
Nox1 expression increased in neurons of ischemia—reperfusion (I/R) brain after MCAO—reperfusion. (a) Representative photomicrographs of western blots for Nox1, Nox2, and β-actin in total lysates of the peri-infarct regions at 6 h, 1 d, 1 w, and 2 w after I/R and control brain. NC: Lysates of the smooth muscle cell of *Nox1* knockout mice were used as a negative control against Nox1 antibody. C: control brain (b) Representative photomicrographs of western blots for activated Rac1 with the active GTPase pull-down assay and Rac1 in total lysates of the peri-infarct regions at 6 h, 1 d, 1 w, and 2 w after I/R and control brain. (c) Signal intensities were measured using Quantity One software and are shown as a percentage of control. β-Actin or Rac1, internal control. Results are presented as the mean ± SEM, n = 4/4. **p<0.01, ***p<0.001 vs sham control. ###p<0.001 vs 6 h, 1 d, 1 w, or 2 w after IR (d) Schematic diagram of the infarct core and the peri-infarct region delineated by Nissl staining at 1 week after MCAO—reperfusion. Black: infarct core; black dotted line: peri-infarct region; gray colored boxes: Nox1 immunostaining observed areas; A: contralateral area; B: ipsilateral area. (e) Representative photomicrograph of Nox1 immunostaining in the peri-infarct regions of I/R brain at 2 weeks. Immunostaining of Nox1 (red) and NeuN (green) in cells of the peri-infarct regions in the cortex. NeuN (green) and Nox1 (red) were visualized in the peri-infarct regions at 2 weeks after I/R. Nox1 expression in neurons is demonstrated as yellow staining after merging green (NeuN) and red (Nox1) images. Scale bars = 30 μm. (f) Counts of Nox1-positive neurons of total neurons. Results are presented as the mean ± SEM, n = 4/4. **p<0.01 vs cells in the contralateral area. (g and h) Immunostaining of Nox1 (red) and CD11b in GFAP in astrocytes (green) or microglia (green) of the peri-infarct regions. Scale bars = 100 μm. Neither astrocytes nor microglia expressed Nox1, as verified by coimmunostaining of Nox1 with GFAP (astrocytes) or CD11b (microglia). contra: contralateral region; ipsi: ipsilateral region.

To determine the expression of the superoxide generator Nox1, we first immunoblotted protein extracts from tissue. Nox1 expression and Rac1 activation in protein extracts taken from the peri-infarct regions (Fig. 1b) significantly increased at 6 h, 1 d, 1 w, and 2 w after MCAO—reperfusion. Nox2 expression increased at 1 d, 1 w, and 2 w after MCAO—reperfusion (Fig. 3a, b, and c). The intensity of Nox1 expression was higher than that of Nox2 expression. To confirm the results of the Nox1 immunoblotting analysis, total lysates of the smooth muscle cells (SMC) of *Nox1* knockout mice were used as a negative control (Fig. 3a and c). In Western blot analysis, Nox1 was not detected in the SMC of *Nox1-* deficient mice. However, Nox2 was expressed constitutively in the SMC of *Nox1-* deficient mice. To localize Nox1 expression in the brain, tissue sections were immunostained for Nox1 and NeuN, GFAP, or CD11b. Nox1 expression was significantly increased in the neurons of the peri-infarct regions (Fig. 3d) at 1 w after MCAO—reperfusion. Neurons immunostained for Nox1 (red) had coexpression of NeuN (green) in the peri-infarct regions (Fig. 3d) indicating neuron-specific expression of Nox1 (Fig. 3e and f).

Neither astrocytes nor microglia expressed Nox1 in the peri-infarct regions, as verified by coimmunostaining of Nox1 with GFAP (astrocytes) or CD11b (microglia) (Fig. 3g and h).

### Adeno-associated virus (AAV)-mediated Nox1 knockdown reduces infarct formation after MCAO in rats

**Fig 4:**
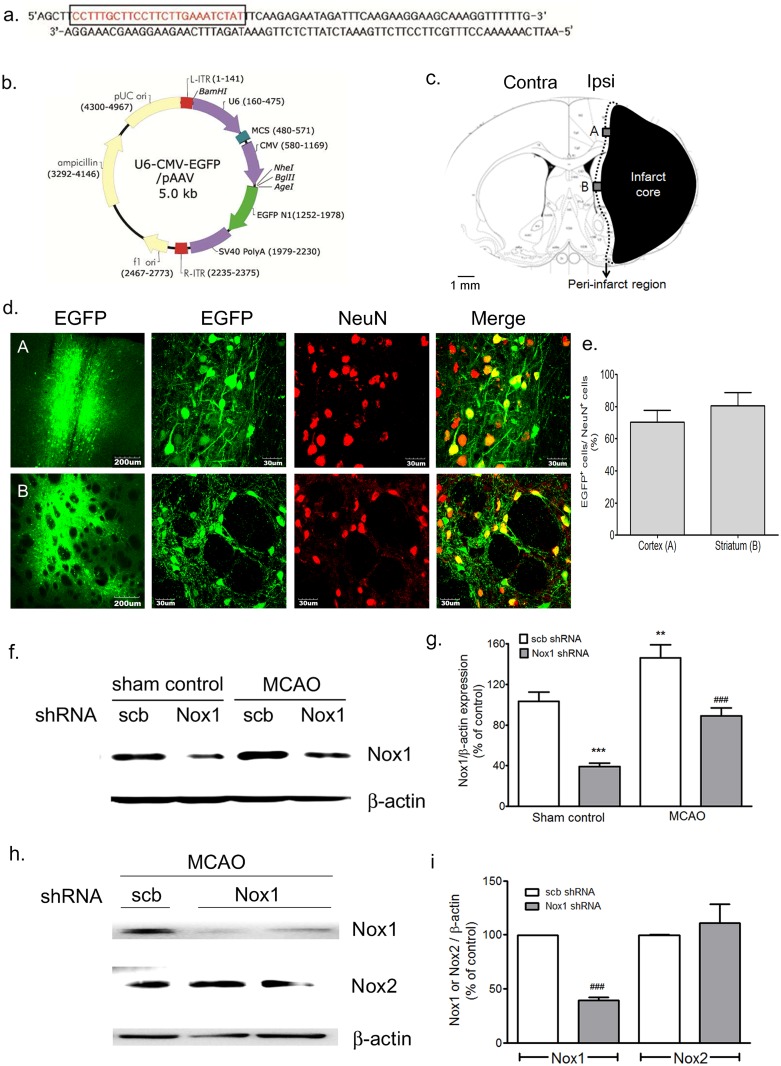
Establishment of U6 promoter-based Nox1 shRNA/AAV vector and identification of the transduction in cells of the peri-infarct regions. (a) Nox1 shRNA sequence. Nox1 shRNA was designed based on the siRNA sequence (boxed red nucleotides) (b) Establishment of the Nox1 shRNA/AAV construct. U6 promoter-driven shRNA expression system was established in the AAV2 vector. EGFP expression is separately controlled by a CMV promoter as a marker for the transduction efficiency. (c) Schematic diagrams of the infarct core and the peri-infarct region delineated by Nissl staining at 2 weeks after MCAO—reperfusion. Black: infarct core; black dotted line: peri-infarct region; gray colored boxes: EGFP expression observed areas; A: peri-infarct area in the cortex; B: peri-infarct area in the striatum. (d) Representative photographs of tissue sections expressed EGFP (green) and stained with the NeuN (red) antibody from rat cortex and striatum tissue taken from rats at 6 weeks after Nox1 shRNA/AAV2 injection. shRNA expression in NeuN^+^ neurons is demonstrated as yellow staining after merging green (EGFP) and red (NeuN) images. (e) EGFP expression levels were measured as cell counts of EGFP expression in NeuN^+^ neurons in the cortex and striatum n = 6/group. (f, g, h, and i) Nox1 knockdown efficiency (f and g) and specificity (h and i) in the cortex and striatum was verified by western blot analysis for Nox1, Nox2, and β-actin performed at 8 weeks after AAV injection. n = 4/group, **p<0.01, ***p < 0.001 vs sham control with scb shRNA, ###p < 0.001 versus MCAO control with scb shRNA. Scb, scramble.

**Fig 5:**
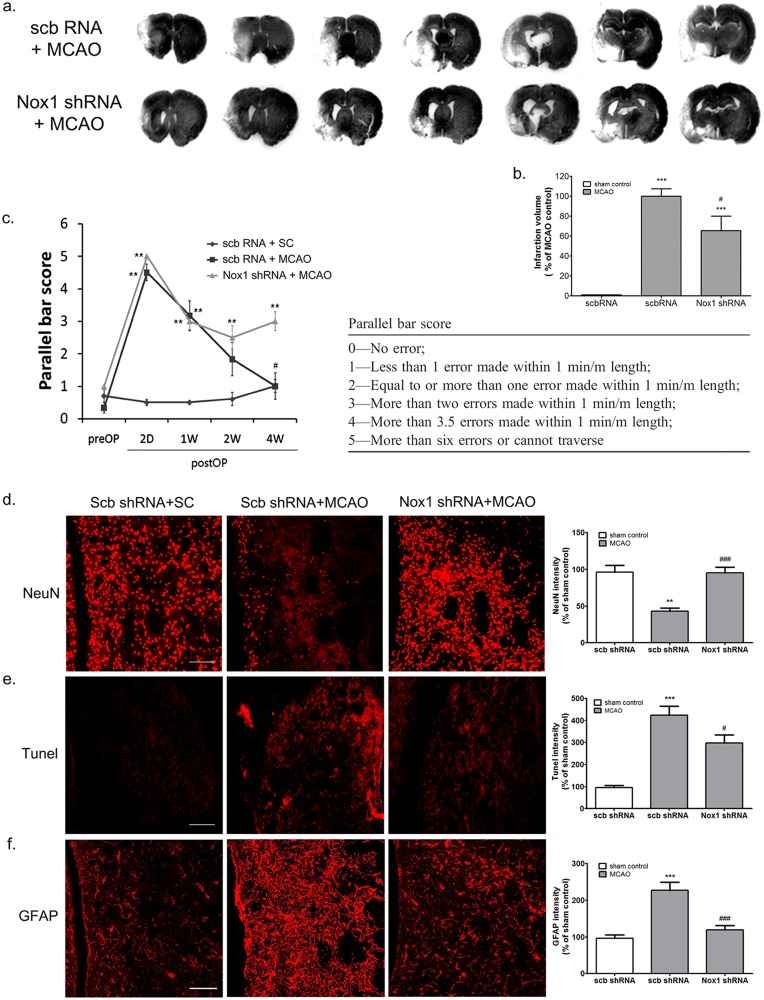
AAV-mediated Nox1 knockdown reduces infarct formation, motor dysfunction, neuronal death, and astrocyte activation at 4 weeks after ischemia—reperfusion. (a) Representative photomicrography of Nissl-stained sections after MCAO with scbRNA or Nox1 shRNA expression. (b) Quantification of infarction size in sham control and MCAO with scbRNA or Nox1 shRNA group. Results are presented as the mean ± SEM, n = 8/8. **p<0.05 vs sham control, ^+^p<0.05 vs MCAO with vector/AAV2 expression. (c) AAV-mediated Nox1 knockdown improved recovery of motor function after MCAO—reperfusion using by parallel bar test. **p<0.01 vs pre-MCAO, # p<0.05 vs MCAO with scbRNA. Results are presented as the mean ± SEM, n = 8/8. Scb, scramble. (d, e, and f) Representative photomicrography and intensity of immunoreactivities of (d) NeuN-, (e) TUNEL-, and (f) GFAP-stained sections after MCAO with scbRNA or Nox1 shRNA expression. Scale bars = 60 μm. **p<0.01, ***p<0.001 vs sham control with scb shRNA, # p<0.05, ###p<0.001 vs MCAO with scb shRNA. Results are presented as the mean ± SEM, n = 6/8.

To investigate the role of Nox1 in MCAO, an AAV2 containing an shRNA targeting Nox1 was delivered stereotaxically into the cortex and striatum (Fig. 4c and d). The vectors expressed enhanced green fluorescent protein (EGFP) separately, as a marker of transduction efficiency (Fig. 4a and b). AAV2 containing the shRNA construct was transferred to neurons in the brain, and efficiently knocked down Nox1 expression. MCAO was performed at 4 w after AAV2 injection; about 70% of NeuN^+^ cells in the cortex and about 80% of NeuN^+^ cells in the striatum were transduced with AAV2 particles, as indicated by the EGFP signal (Fig. 4d and e). Nox1 knockdown efficiency in the cortex and striatum (Fig. 4c) was verified by western blot analysis at 8 w after AAV2 injection (Fig. 4e and 4f). We confirmed that the Nox1 shRNA has no effect on other isoforms, and specifically, Nox2 expression (Fig. 4g and h). Nox1 expression was completely blocked by Nox1 shRNA. However, Nox2 expression was not altered by Nox1 shRNA. An injection of Nox1 shRNA viral particles (Nox1 shRNA) in the cortex and striatum 4 w before MCAO-reperfusion significantly diminished the resulting infarction volume. Infarction volume was measured by Nissl staining of brain tissues on day 28 after MCAO (Fig. 5a). The infarction volume from MCAO-reperfusion in the rats with and without Nox1 knockdown was compared. Transient MCAO (90 min) and reperfusion (4 w) resulted in an infarct in the ipsilateral half of the brain encompassing the cerebral cortex and striatum from—5.2 to +2.8 from bregma. The total infarct volume from MCAO-reperfusion was significantly different between the rats treated with scrambled shRNA viral particles (scbRNA) (100%) and those treated with Nox1 shRNA particles (Fig. 5b).

### AAV-mediated Nox1 knockdown enhanced functional recovery after MCAO

Functional recovery was determined using parallel bar tests in rats at—1, 2, 7, 14, and 28 d after MCAO. Parallel bar scores were significantly different between the control group and the Nox1 knockdown group on day 28 after MCAO (P<0.05, Fig. 5c).

### AAV-mediated Nox1 knockdown reduced neuronal death and astrocyte activation after MCAO

The number of NeuN-positive neurons in Nox1 shRNA-expressing rats was significantly less than in rats without transduction (Fig. 5d). In similar experiments, Nox1 knockdown significantly reduced TUNEL-positive neuronal death elicited by MCAO—reperfusion, indicating that inhibition of Nox1 expression attenuated MCAO—reperfusion-elicited neuronal apoptosis (Fig. 5e). GFAP-positive astrocyte activation was significantly decreased in Nox1 shRNA-expressing rats (Fig. 5f).

### Nox1 knockdown improved the survival and differentiation of progenitor cells in the SVZ after cerebral ischemia

**Fig 6:**
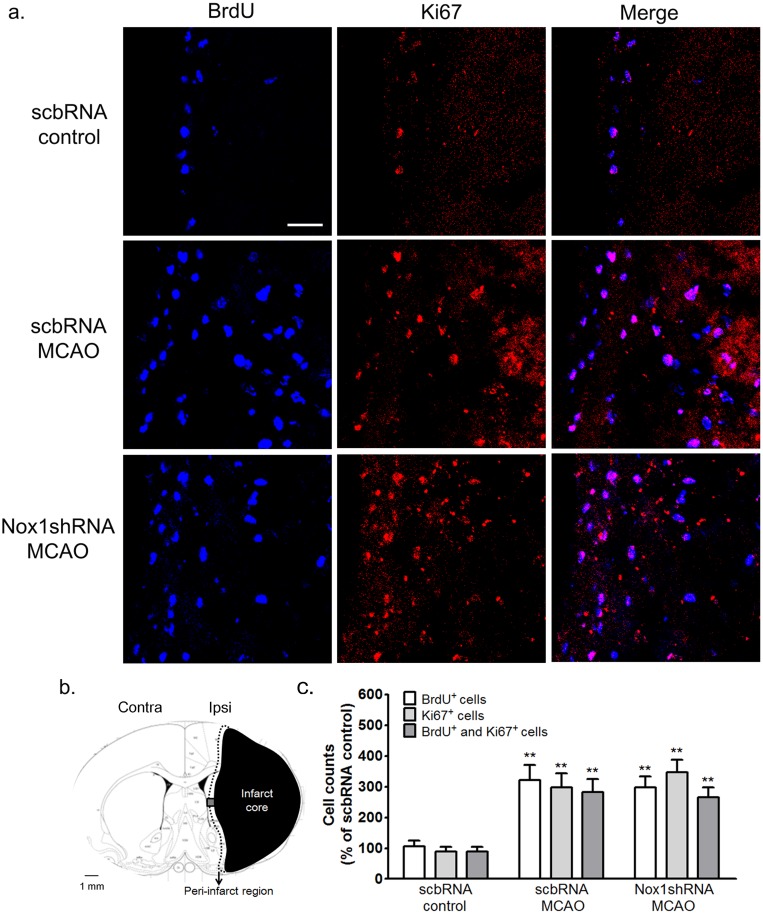
Nox1 knockdown did not affect the proliferation of progenitor cells in the SVZ after cerebral ischemia. (a) Representative photomicrograph of BrdU or Ki67 immunostaining showing proliferating cells in the SVZ area at 2 weeks after ischemia—reperfusion. BrdU and Ki67 coexpression in the SVZ is demonstrated as purple staining after merging red (Ki67) and blue (BrdU) images. Scale bars = 30 μm. (b) Schematic diagrams of the infarct core and the peri-infarct region delineated by Nissl staining at 2 weeks after MCAO—reperfusion. Black: infarct core; black dotted line: peri-infarct region; gray colored boxe: BrdU and Ki67 immunostaining observed areas (c) BrdU- and Ki67-stained cells were counted in the ischemia—reperfusion brain. Results are presented as mean ± SEM, n = 6/6. **p<0.01 vs the scbRNA control. scbRNA: scramble shRNA; control: sham control.

**Fig 7:**
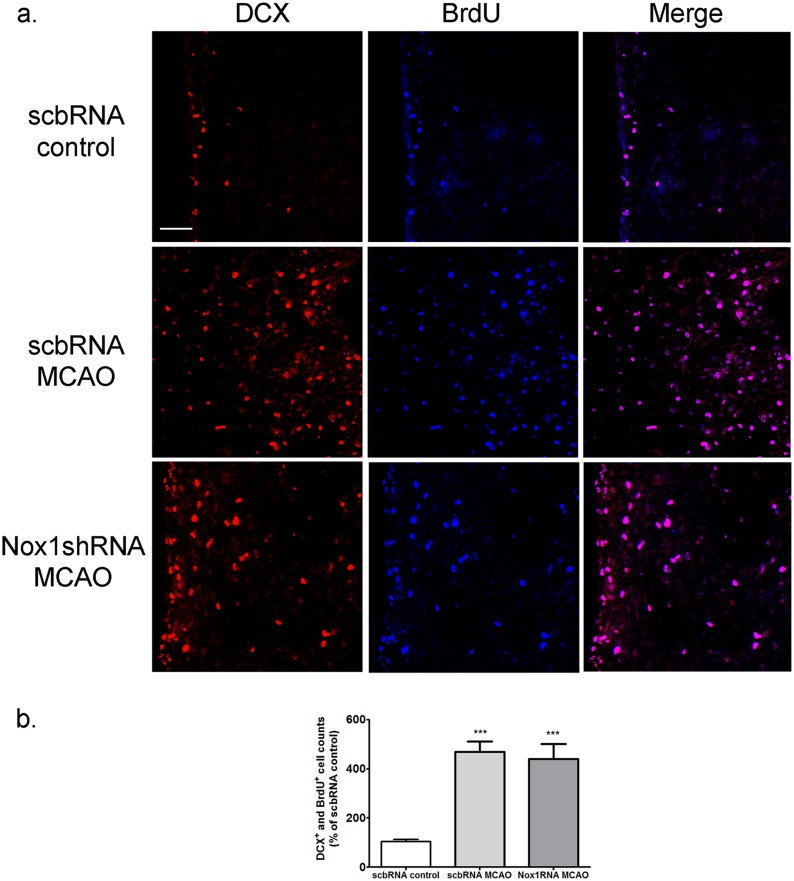
Nox1 knockdown did not affect the proliferation of neural progenitor cells in the SVZ after cerebral ischemia. (a) Representative photomicrograph of DCX or BrdU immunostaining showing proliferating cells in the SVZ area at 2 weeks after ischemia—reperfusion. DCX and BrdU coexpression in the SVZ is demonstrated as purple staining after merging red (DCX) and blue (BrdU) images. Scale bars = 50 μm. (B) DCX- and BrdU- stained cells were counted in the ischemia—reperfusion brain. Results are presented as mean ± SEM, n = 6/6. **p<0.01 vs scbRNA control. DCX: doublecortin; scbRNA:scramble shRNA.

**Fig 8:**
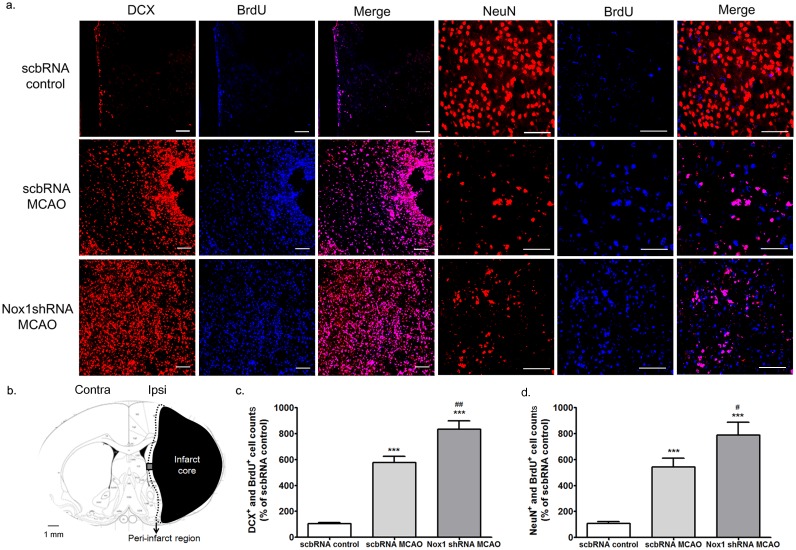
Nox1 knockdown improved the differentiation of progenitor cell in the ischemic region after cerebral ischemia. (a) Representative photomicrograph of BrdU (blue) and DCX (red) double immunostaining showing progenitor cells in the peri-infarct region at 2 weeks after ischemia—reperfusion and BrdU (blue) and NeuN (red) double immunostaining showing matured newborn neurons in the peri-infarct region at 4 weeks after ischemia—reperfusion. Scale bars = 50 μm. (b) Schematic diagrams of the infarct core and the peri-infarct region delineated by Nissl staining at 2 weeks after MCAO—reperfusion. Black: infarct core; black dotted line: peri-infarct region; gray colored box: BrdU, DCX, and NeuN immunostaining observed areas (c) BrdU and DCX double-stained cells were counted in the peri-infarct region after cerebral ischemia. Results are presented as the mean ± SEM, n = 6/6. **p<0.01 vs scbRNA control. ## p<0.01 vs scbRNA MCAO (d) BrdU and NeuN double-stained cells were counted in the peri-infarct region after cerebral ischemia. Results are presented as the mean ± SEM, n = 6/8. ***p<0.001 vs scbRNA control, # p<0.05 vs scbRNA MCAO. scbRNA: scramble shRNA.

Sham-operated rats and rats with MCAO received an injection of the cell proliferation marker BrdU (50 mg/kg, intraperitoneally, twice daily) from days 5 to 14. Rats were killed on days 14 or 28. Most BrdU-positive (BrdU^+^) cells were localized to the SVZ (Fig. 6a). The number of BrdU^+^ cells was seen to be increased on postischemic day 14 in the rat SVZ and the peri-infarct resions (Fig. 6b). The increase was statistically significant (Fig. 6a and c). BrdU incorporation could also reflect a DNA repair process, as distinct from cell proliferation. We therefore determined immunoreactivity for Ki67, a nuclear protein closely associated with cell mitosis [8]. Immunohistochemistry for Ki67 showed a similar distribution and increase to BrdU immunoreactivity (Fig. 6a and c). The BrdU^+^/Ki67^+^ cells were on average 90 ± 12.5% of all BrdU^+^ cells (Fig. 6c). We also found that the expression of Ki67 and uptake of BrdU was similarly regulated and colocalized the ipsilateral SVZ and the peri-infarct regions of MCAO rats treated with scb shRNA/AAV or Nox1 shRNA/AAV particles before MCAO (Fig. 6a and c). To determine the effect of Nox on ischemia-induced cell proliferation, BrdU and doublecortin (DCX) double-labeling methods were used to detect cells proliferating in the ipsilateral SVZ of rats at 14 days after MCAO-induced ischemia. The BrdU- and DCX-positive cells were increased in the ipsilateral SVZ following cerebral ischemia compared with the contralateral SVZ following MCAO. No significant difference in the number of BrdU^+^ cells in the ipsilateral SVZ was observed between the rats treated with scbRNA and rats treated with Nox1 shRNA before MCAO (Fig. 7). However, the number of BrdU^+^ and DCX^+^ double-labeled cells in the ipsilateral peri-infarct region of rats treated with Nox1 shRNA was significantly increased compared with rats treated with scbRNA before MCAO (Fig. 8). These findings suggest that Nox1 knockdown increased the localization of ischemia-induced progenitor neurons in the MCAO ischemic region.

Next, to examine the effect of Nox1 on the differentiation of progenitor cells and the survival of newborn cells in ischemia, BrdU- and NeuN-labeling methods, and BrdU- and active caspase-3-labeling methods were used to detect differentiated cells and apoptotic newborn cells, respectively, in the ipsilateral peri-infarct regions in ischemic rats at 4 w after MCAO. The number of BrdU and NeuN double-labeled cells in the ipsilateral peri-infarct region of rats treated with Nox1 shRNA was significantly increased compared with rats treated with scbRNA before MCAO (Fig. 8).

**Fig 9:**
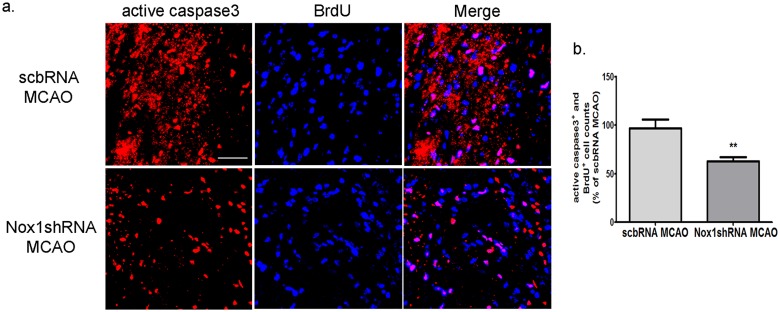
Nox1 knockdown improved the survival of newborn cells in the penumbra after cerebral ischemia. (a) Representative photomicrograph of BrdU (blue) and active caspase-3 (red) double immunostaining showing apoptotic newborn cells in the penumbra area at 4 weeks after ischemia—reperfusion. BrdU and active caspase-3 coexpression in the penumbra is demonstrated as purple staining after merging red (active caspase-3) and blue (BrdU) images. Scale bars = 50 μm. (b) BrdU and active caspase-3 double-stained cells were counted in ischemia—reperfusion brain. Results are presented as the mean ± SEM, n = 6/8. ** p<0.01 vs scbRNA MCAO. scbRNA: scramble shRNA.

The number of BrdU and active caspase-3 double-labeled cells in the peri-infarct region of rats treated with Nox1 shRNA was significantly decreased in comparison with rats treated with scbRNA before MCAO (Fig. 9a and b).

These results indicate that Nox1 knockdown increases newborn cell survival and promotes progenitor cell differentiation into mature neurons in the MCAO ischemic region.

## Discussion

In the present study, we showed that Nox1 mediates oxidative stress, neuronal death, and activation of astrocytes in rats with MCAO. We demonstrated, for the first time to our knowledge, that the expression of the Nox1 isoform is elevated in the peri-infarct region after stroke.

The peri-infarct region is only viable for a limited time, but as long as energy metabolism and membrane function are preserved, the injury is fundamentally reversible. Changes in the viability of the peri-infarct region are therefore of critical importance to assess the progression of tissue damage and to determine the window of opportunity for therapeutic interventions [23].

Accumulation of the Nox1 complex in the peri-infarct region, production of ROS, and nuclear DNA damage were all found in neurons of the I/R-injured brain. These findings indicate that Nox1-mediated oxidative damage may serve as a critical upstream process in neurodegeneration of the peri-infarct region.

There is increasing evidence that generation of ROS in the CNS involves the Nox family of NADPH oxidases [24]. Nox enzymes are not restricted to microglia (i.e. brain phagocytes), but are also expressed in neurons, astrocytes, and the neurovascular system. Nox enzymes are involved in CNS development, neural stem cell biology, and the function of mature neurons. While Nox2 appears to be a major source of pathological oxidative stress in the CNS, other Nox isoforms might also be of importance, e.g. Nox4 in stroke [24].

A first proof of concept for Nox2 as a principal source of ROS in stroke was provided in 1997 when Walder et al. showed strong protection for the damage of transient MCAO in Nox2-deficient mice [25], raising strong interest in targeting Nox2 as a potential therapy for stroke. Since then, this paradigm has been tested in numerous models using small molecules and Nox1-, Nox2-, and Nox4-deficient mice, resulting in different outcomes and leading to disputes whether Nox2 [26] or Nox4 [7] isoform is the key pharmacological target for stroke [24]. Although few studies have reported a role for Nox1 in experimental stroke, we recently reported a moderate reduction in lesion volume and cerebral edema, and ameliorated BBB leakage with relatively mild ischemic damage after the knockout of Nox1 [9]. Studies examining the role of Nox2 have demonstrated activation via N-methly-D-aspartate (NMDA) receptors [24, 27, 28]. However, the role of neuronal Nox1 activation in stroke is not well understood. Mitochondria are involved in Nox1 expression in nonneuronal cells [29–31]. In serum-deprived 293 cells, mitochondrial ROS at the early phase contributes to Nox1 induction that is responsible for the later phase of ROS accumulation, culminating in cell death [32]. Studies of osteosarcoma cells with mitochondrial gene knockout (q0) revealed that the inactivation of mitochondrial genes leads to downregulation of Nox1 and that the transfer of wild-type mitochondrial genes can restore Nox1 expression [31]. We found that newly discovered molecular mechanisms underlie oxidative stress-mediated dopaminergic neuronal death, in which matrix metalloproteinase 3 activation is a key upstream event that leads to mitochondrial ROS, Nox1 induction and eventual dopaminergic neuronal cell death (data not shown). To understand these mechanisms better, we need to determine the mechanism of Nox1 activation and regulation in neuronal damage after stroke.

Our results showed that increased infarction size, neuronal cell death using NeuN^+^ cell counts and TUNEL staining, and activation of astrocytes were significantly reduced by Nox1 knockdown in the peri-infarct region. Moreover, AAV-mediated Nox1 knockdown enhanced functional recovery after MCAO.

Nox1-mediated superoxide generation requires other components, including Rac1 activation, and Noxo1 and Noxa1, which are homologues of p47phox and p67phox, respectively [33]. Rac1, a small Rho GTPase, is a protein that is important for the activation of Nox1-derived superoxide generation [33, 34]. To activate the NADPH oxidase system, activated Rac1 forms a Nox1 enzyme complex in conjunction with Noxo1 and Noxa1 [35]. In the present study, we found that neurons in the peri-infarct region were equipped with Noxo1 and Noxa1; moreover, MCAO-mediated Rac1 activation was observed.

Acute brain injury, such as cerebral ischemia, increases the ratio of neurogenesis in both the SVZ and SGZ of adult rodents [36, 37], which suggests that the activation of neural stem cells that migrate into lesion regions may be involved in self-repair to compensate for the local cell loss [37–39]. Unfortunately, most of new neurons in the adult brain are not preserved. Following ischemic stroke, 80% of new neurons die within 2 to 6 w after stroke [40, 41], which indicates that new neurons that originate from precursor cells are susceptible to brain injury [42]. Accordingly, strategies to enhance the differentiation of progenitor cells into neuroblasts and improve the survival of new neurons after brain injury are beneficial [43]. The survival of new neurons is dependent on the surrounding milieu, which is prominently altered after cerebral ischemia, usually through the accumulation of inflammatory factors, mitotic factors, and toxins [44, 45]. Although the mechanism remains unclear, the accumulation of inflammatory factors and toxins has recently been proposed to be part of the neurogenic environment that affects progenitor cell proliferation, differentiation, and survival [37]. Therefore, we investigated the effect of Nox1 knockdown on progenitor cell proliferation, differentiation, and survival in the peri-infarct region. We found that Nox1 knockdown improved the survival and differentiation of progenitor cells in the SVZ after cerebral ischemia.

Our data suggest that Nox1 may be involved in oxidative stress and neuronal death in the peri-infarct region after stroke. Moreover, inhibition of Nox1 in the peri-infarct region can improve neurological dysfunction and enhance the survival of new neurons in the peri-infarct region by inhibiting apoptosis and downregulation of superoxide generation after transient cerebral ischemia in rats. Therefore, Nox1 may serve as a potential therapeutic target for brain repair after stroke.
